# Keratitis by *Fusarium temperatum*, a novel opportunist

**DOI:** 10.1186/s12879-014-0588-y

**Published:** 2014-11-12

**Authors:** Abdullah M S Al-Hatmi, Alexandro Bonifaz, G Sybren de Hoog, Leticia Vazquez-Maya, Karla Garcia-Carmona, Jacques F Meis, Anne D van Diepeningen

**Affiliations:** CBS-KNAW Fungal Biodiversity Centre, Utrecht, 3508 AD The Netherlands; Institute of Biodiversity and Ecosystem Dynamics, University of Amsterdam, Amsterdam, The Netherlands; Directorate General of Health Services, Ibri Hospital, Ministry of Health, Muscat, Oman; Hospital General de México, O.D, Mexico City, Mexico; Peking University Health Science Center, Research Center for Medical Mycology, Beijing, China; Sun Yat-Sen Memorial Hospital, Sun Yat-Sen University, Guangzhou, China; Shanghai Institute of Medical Mycology, Changzheng Hospital, Second Military Medical University, Shanghai, China; Basic Pathology Department, Federal University of Paraná State, Curitiba, Paraná Brazil; King Abdulaziz University, Jeddah, Saudi Arabia; Department of Medical Microbiology and Infectious Diseases, Canisius Wilhelmina Hospital, Nijmegen, The Netherlands; Department of Medical Microbiology, Radboud University Medical Center, Nijmegen, The Netherlands

**Keywords:** Keratitis, Fusarium temperatum, Maize, Molecular phylogenetics, Infection

## Abstract

**Background:**

*Fusarium* species are among the most common fungi present in the environment and some species have emerged as major opportunistic fungal infection in human. However, in immunocompromised hosts they can be virulent pathogens and can cause death. The pathogenesis of this infection relies on three factors: colonization, tissue damage, and immunosuppression. A novel *Fusarium* species is reported for the first time from keratitis in an agriculture worker who acquired the infection from plant material of maize. Maize plants are the natural host of this fungus where it causes stalk rot and seeding malformation under temperate and humid climatic conditions. The clinical manifestation, microbiological morphology, physiological features and molecular data are described.

**Methods:**

Diagnosis was established by using polymerase chain reaction of fungal DNA followed by sequencing portions of translation elongation factor 1 alpha (TEF1 α) and beta-tubulin (BT2) genes. Susceptibility profiles of this fungus were evaluated using CLSI broth microdilution method.

**Results:**

The analyses of these two genes sequences support a novel opportunist with the designation *Fusarium temperatum*. Phylogenetic analyses showed that the reported clinical isolate was nested within the *Fusarium fujikuroi* species complex. Antifungal susceptibility testing demonstrated that the fungus had low MICs of micafungin (0.031 μg/ml), posaconazole (0.25 μg/ml) and amphotericin B (0.5 μg/ml).

**Conclusion:**

The present case extends the significance of the genus *Fusarium* as agents of keratitis and underscores the utility of molecular verification of these emerging fungi in the human host.

**Electronic supplementary material:**

The online version of this article (doi:10.1186/s12879-014-0588-y) contains supplementary material, which is available to authorized users.

## Background

Fungal keratitis was first reported by Theodor Leber in 1879 in a farmer who had an eye trauma due to blades used for cutting wheat. Today the infection is known to occur worldwide, particularly in warmer climates, and is caused by a large diversity of fungal species. In temperate regions, fungal keratitis is most commonly caused by *Fusarium species*[1]. Under (sub) tropical conditions, filamentous fungi are prevalent as causes of infection. Particularly *Fusarium* and *Aspergillus* predominate, with up to one-third of cases of traumatic keratitis [2],[3]. On a global scale, fusariosis is one of the most common causes of fungal corneal ulcers [4]-[6].

Keratitis caused by *Fusarium* is a serious infection and occurs especially among farmers and workers with agricultural occupations. Corneal abrasions occur commonly during harvest, when labor handling decayed and dried plant products is a major risk factor for ocular trauma [7]-[9]. Fungi are one of the possible causes of keratitis and are differentially susceptible to commonly used antifungals. Therefore misdiagnosis potentially leads to visual loss and devastating ocular damage if the infection remains untreated [10].

*Fusarium* is a genus of more than 200 species of molds that are widely distributed in soil, on terrestrial plants, in plant debris and on other organic substrates. Numerous agents of diseases of plants and cold-blooded animals are known [11], but only a few have been recognized as causing infections in humans [12]. *Fusarium* infections in immunocompetent hosts are mostly associated with superficial mycosis such as onychomycosis and keratitis; the first reported case of a *Fusarium* eye infection dates back to 1958 [13]. Recently, deep and systemic infections are observed in immunocompromised patients, with increasing frequency [14].

Here we present an extraordinary case of keratitis in a worker who acquired the infection from plant material of maize. The case is worth reporting not only by its rareness but also its unusual infection in a human. In the present report, this case was initially ascribed to *Fusarium oxysporum* based on the morphological characters. Since morphological studies are insufficient to determine the correct taxonomic position at the species level in *Fusarium*, a multilocus DNA sequence study followed by phylogenetic analysis was applied to identify the agent of this case. As a result, *Fusarium temperatum* is reported as a new causative agent of human keratitis.

### Case report

**Figure 1:**
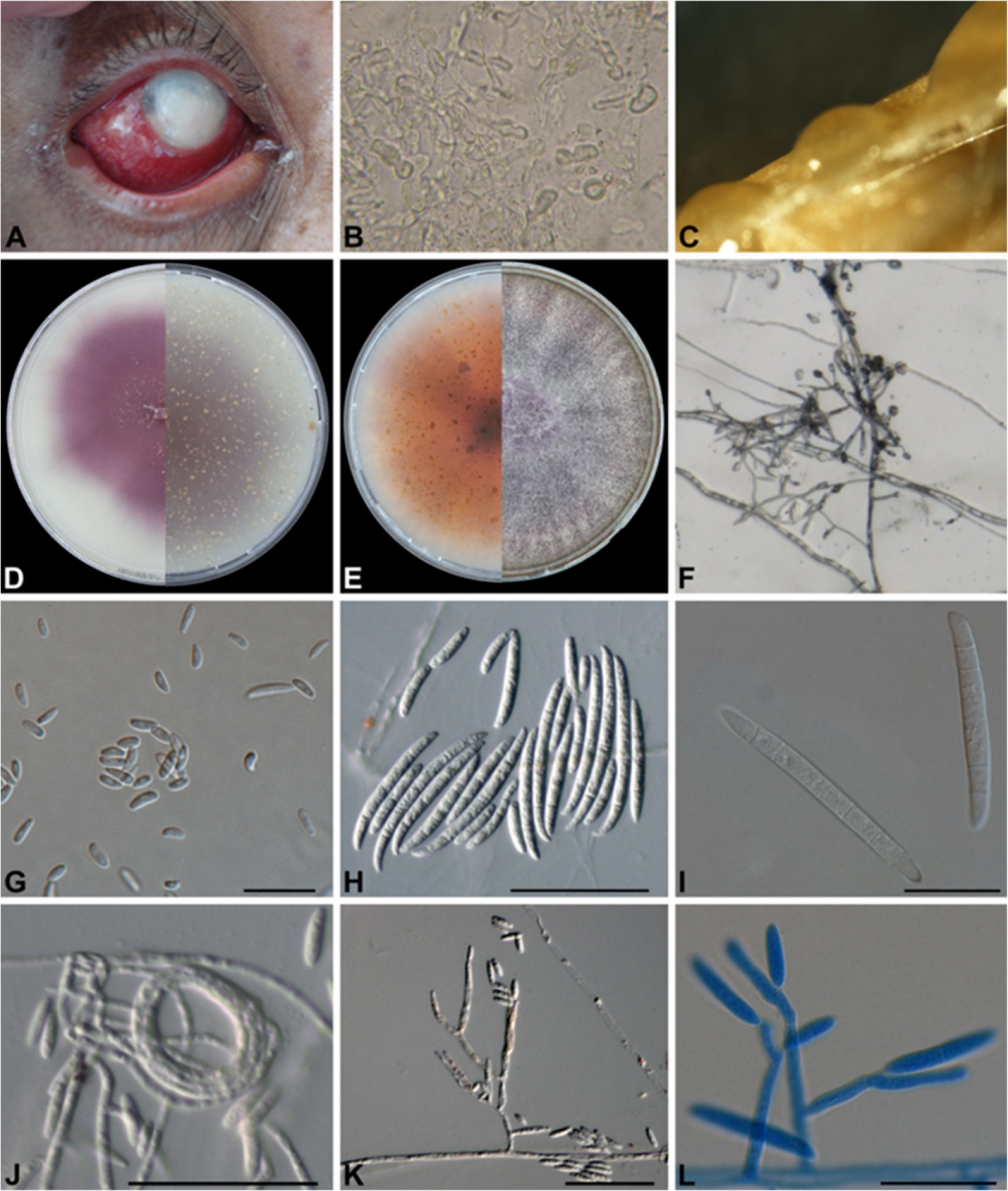
**Morphological description of** ***Fusarium temperatum.*** **(A)** Slit lamp photograph showing infected cornea involving regions of sclera; **(B)** KOH mount of the scraping material showing fungal hyaline and nonseptate hyphae (magnification, ×40); **(C)** Sporodochia present in yellowish orange on CLA; **(D)** Growth of the isolate *F. temperatum* on OA, agar pigmentation ranges from colorless to dark purple on; reverse pigmentations in light pink; **(E)** Growth of isolates on PDA at 25°C; **(F)** *In situ* conidiophores with false heads; **(G)** Microconidia on CLA; **(H-I)** Macroconida; **(J)** Coild hyphae; **(K-L)** Monophialidic and polyphialidic conidiogenous cells. All scale bars, 10 μm.

An agriculture worker presented with a corneal ulceration. The disorder had started 12 days earlier when patient suffered from a trauma in the eye with maize plant materials during harvest. Before presenting to the hospital, the patient was seen by a local practitioner who prescribed neomycin, polymyxin B, phenylephrine and dexamethasone eye drops for eight days, supposing that a bacterial infection was concerned. On presentation, visual acuity was 20/50 by using Snellen chart. Slit-lamp examination of the right eye showed a 12 mm white-yellowish central corneal epithelial defect with irregular and raised edges, along with intense hyperemia of the conjunctiva, photophobia and pain (Figure 1A).

Corneal scrapings for direct microscopic examination with 10% potassium hydroxide demonstrated multiple irregular, septate, hyaline hyphae (Figure 1A). Samples were inoculated onto Sabouraud’s glucose agar (SGA) plates and incubated for 5 days at 28°C. Fluffy, pink-violet colonies rapidly developed, which microscopically revealed abundant sickle-shaped, septate macroconidia (4–5 μm in length) in addition to microconidia (2–3 μm in length). On the basis of culture and microscopy, the fungus was provisionally identified as *F. oxysporum*.

Antifungal treatment was initiated with topical natamycin 5% (Laboratorios Grin, Distrito Federal, Mexico) ophthalmic solution, as follows: 2 drops each hour during eight days, then one drop each 4 hours, in addition to itraconazole 200 mg daily. Noteworthy improvement was achieved, and the patient did not return for follow up visits.

## Methods

### Ethics statement

The study protocol was approved by the Scientific and Ethics Committees of the Hospital General de México (approval number DIC/12/102/3/23) and was performed in accordance with the ethical principles described in the 1964 Declaration of Helsinki. Informed written consent was obtained from the patient prior to their inclusion in the study.

### Clinical specimen

Corneal scrapings were collected for microbiological studies from a patient seen in Hospital General de México, O.D, Mexico City, Mexico.

### Fungal isolation

Corneal scrapings were inoculated onto Sabouraud’s glucose agar (SGA) plates and incubated for 5 days at 28°C. Subcultures of the causative agent were deposited in the reference collection of the CBS-KNAW Fungal Biodiversity Centre, Utrecht, The Netherlands under accession number CBS 135540, where further identification was undertaken.

### Morphology

The fungus grew on culture plates of malt extract ager (MEA; Oxoid, U.K.), oatmeal agar (OA; home-made at CBS), potato dextrose agar (PDA; Oxoid), synthetic nutrient agar (SNA) [15], and carnation leaf agar (CLA) [16]. Culture plates were incubated in the dark for one week at 25°C. Microscopic mounts in lactic acid with cotton blue were made from cultures grown on a PDA plate. Slide cultures were observed after 5 days of incubation at 25°C. Slides were examined and measured with a light microscope (Nikon Eclipse 80i), and pictures were taken using a Nikon digital-sight DS-5 M camera attached to the microscope.

### Physiology

Cardinal growth temperatures were determined on PDA with isolates incubated in the dark for one week at 25, 27, 30, 33, 35, 36, 37 and 40°C.

### DNA extraction

DNA was extracted following the Quick CTAB protocol. 1–10 mm^3^ fungal material was transferred to two mL screw-capped tubes filled with 490 μL CTAB-buffer 2 × and 6–10 acid-washed glass beads. 10 μL Proteinase K were added and mixed thoroughly on a MoBio vortex for 10 min. 500 μL Chloroform: isoamylalcohol (24:1) was added and shaken for 2 min after incubation for 60 min at 60°C. Tubes were centrifuged for 10 min at 14,000 r.p.m. The supernatant was collected in a new Eppendorf tube. To ~400 μL DNA sample 2/3 vol (~270 μL) of ice-cold iso-propanol was added and centrifuged again at 14,000 r.p.m. for 10 min and the upper layer was dissolved in 1 mL ice-cold 70% ethanol. Tubes were centrifuged again at 14,000 r.p.m. for 2 min, air-dried and re-suspended in 50 μL TE-buffer. The quality of genomic DNA was verified by running 2–3 μL on a 0.8% agarose gel. DNA was quantified with a NanoDrop 2000 spectrophotometer (Thermo Fisher, Wilmington, U.S.A.). Samples were stored at -20°C until use.

### DNA amplification and sequencing

Two gene regions were amplified directly from the genomic DNA for multilocus sequence typing. The primer pairs for the genes were EF1 and EF2 [17], BT-2a [18] and BT-2b [19] (primers listed in Table 1). PCR reaction mixture (12.5 μL final vol) contained 10 × PCR buffer 1.25 μL, water 7.5 μL, dNTP mix (2.5 mm) 0.5 μL, 0.25 μL of each primer (10 pmol), *Taq* polymerase (5 U/μL) 0.05 μL, DMSO 0.7 μL, and template DNA (100 ng/μL) 1 μL. Amplification was performed in an ABI PRISM 2720 (Applied Biosystems, Foster City, U.S.A.) thermocycler as follows: 95°C for 4 min, followed by 35°Cycles consisting of 95°C for 45 sec, 52°C for 30 sec and 72°C for 2 min, and a delay at 72°C for 7 min. Annealing temperature was changed to 58°C for the BT2 gene. PCR products were visualized by electrophoresis on a 1% (w/v) agarose gel. Amplicons were purified using exoSAP. Both strands of the PCR fragments were sequenced with the above-mentioned primers. The ABI PrismH Big DyeTM Terminator v. 3.0 Ready Reaction Cycle Sequencing Kit (Applied Biosystems) was used for sequencing PCR. Sequences were determined with an ABI PRISM™ 3,100 Genetic Analyzer (Applied Biosystems). Sequencing PCR was performed as follows: 1 min at 95°C, followed by 30°Cycles consisting of 10 sec at 95°C, 5 sec at 50°C and 2 min 60°C. Reactions were purified with Sephadex G-50 fine (GE Healthcare Bio-Sciences, Uppsala, Sweden) and sequencing was done on an ABI 3730XL automatic sequencer (Applied Biosystems) with ABI PRISM BigDyeTM terminator cycle sequencing kit.

**Table 1:** **PCR primers used for amplification**

Locus	Primers	primer sequence (5’-3’)	Amplicon size, bp	Ref
TEF1α	EF1	ATGGGTAAGGARGACAAGAC	600	[17]
	EF2	GG ARGTACCAGTSATCATGTT		[17]
BT2	BT-2a	GGTAACCAAATCGGTGCTGCTT	500	[18]
	BT-2b	TTACGTCCCTGCCCTTTGTA		[19]

### Phylogenetic analyses

A consensus sequence was computed from the forward and reverse sequences with SeqMan from the Lasergene package (DNAstar, Madison, WI). Thirty eight sequences of species of the *Fusarium fujikuroi* species complex (FFSC) were included and retrieved from GenBank, including two sequences of the *F. oxysporum* species complex (FOSC) for TEF1 α and BT2 markers as an outgroup (Additional file 1: Table S1). The sequences were aligned using MAFFT v. 7.127 (http://mafft.cbrc.jp), followed by manual adjustments with MEGA v. 5.2. A combined alignment was constructed for both TEF1 α and BT2 markers. The best-fit model of evolution was determined by ModelTest v. 0.1.1. Bayesian analysis was performed with MrBayes v. 3.1.2. Four MCMC chains were run simultaneously for 1 × 10^7^ generations. Bayesian phylogenetic tree was constructed. Sequences of CBS 135540 were deposited in GenBank under the accession numbers [GenBank:KF956084] for TEF-1α and [GenBank:KF956080] for BT2 (Additional file 1: Table S1).

### Antifungal susceptibility

Antifungal susceptibility testing (AFST) of CBS 135540 was performed by the CLSI broth microdilution method, M38-A2 [20]. The antifungals tested were amphotericin B (Sigma, St. Louis, MO), fluconazole (Pfizer, Groton, CT), itraconazole (Janssen Pharmaceutica, Tilburg Netherlands), voriconazole (Pfizer), posaconazole (Merck, Whitehouse Station, NJ), isavuconazole (Basilea Pharmaceutica, Basel, Switzerland), micafungin (Astellas, Ibaraki, Japan), anidulafungin (Pfizer) and natamycin (DSM, Delft, the Netherlands). For the broth microdilution test, RPMI 1640 medium with glutamine without bicarbonate (Sigma) buffered to pH 7 with 0.165 mol/liter 3-N-morpholinepropanesulfonic acid (Sigma) was used. Isolates were grown on PDA for 5 days and incubated at 25°C and for sporulation. Final inoculum was adjusted to a density of 1.0 to 5.0 × 10^4^ hyphal fragments/spores per ml by adjusting an optical density of 0.13 to 0.18 at 530 nm using a spectrophotometer. Drug-free and mold-free controls were included, and microtiter plates were incubated at 35°C for 72 to 96 h. Three reference strains. *Paecilomyces variotii* ATCC 22319, *Candida krusei* ATCC 6258 and *Candida parapsilosis* ATCC 22019 were included as quality controls. The MIC endpoints were read visually, which, for azoles and amphotericin B, were defined as the lowest concentration at which there was 100% inhibition of growth compared with the drug-free control wells. For echinocandins, minimal effective concentrations (MEC) were defined as the lowest concentration of drug that led to the growth of small, rounded, and compact hyphal forms.

## Results

### Morphology

The clinical isolates grew and sporulated well on PDA, OA, CLA and SNA at 25, 27, 30 and 33°C and growth was apparent within 2 days on all agar plates. Yellowish orange sporodochia were produced on CLA (Figure 1C). Agar pigmentation ranged from colorless to dark purple on OA; pigmentation of colony reverse was in shades of light pink (Figure 1D). Aerial mycelium was cottony, initially white, becoming pinkish white, turning violet in the colony center in a later stage. Subsequently, colonies spread rapidly, filling the culture plate within 1 week (Figure 1E). Conidiophores in the aerial mycelium were erect, branched, terminating in 1–3 phialides (Figure 1F). On SNA with filter paper, colonies were colorless, later changing the color of the filter papers to pale pink. Microconidia were oval, abundant, grouped in masses; hyaline and non-septate (Figure 1G). Macroconidia hyaline, with 3–6 (mostly 4–5) septa, slender, slightly falcate, with a beaked, curved apical cell and a foot-like basal cell with a thin cell wall (Figure 1H, I). Polyphialides and monophialides were observed (Figure 1K, L). Chlamydospores were not found over 10 days of incubation.

### Physiology

**Figure 2:**
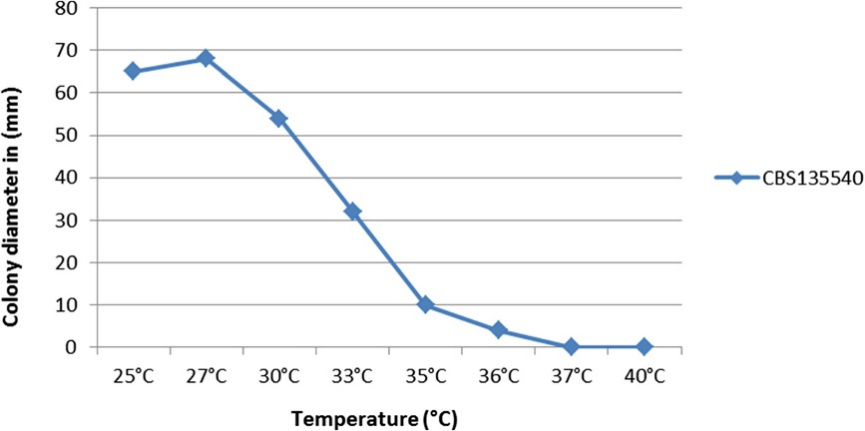
**Average growth of** ***Fusarium temperatum*** **.** Colony diameters (mm) at different temperatures ranging from 25°C to 40°C, measured after 5 days of incubation on 2% MEA, were calculated for *F. temperatum,* CBS135540.

Cardinal growth temperatures tests showed optimal development at 25 − 27°C (Figure 2), with a maximum growth temperature at 36°C. No growth was observed at 37 and 40°C. 37°C proved to be fungistatic, but regrowth was observed after incubation at 25°C. Colonies on PDA attained a diameter of approximately 65 mm, and those on OA covered the entire agar surface after 5 days at 25°C. Colonies attained a diameter of about 68 mm at 27°C in the dark on PDA. Colonies on PDA plates incubated at 33°C showed slow growth and attained a diameter of about 32 mm after 5 days.

### Phylogeny

BT2 and TEF-1α partial genes (NCBI JX987074.1 for TEF-1α and KC964140.1 for BT2) were used for identification of clinical isolate CBS 135540. Both genes possessed enough polymorphism, and therefore, were excellent markers with 99–100% accuracy for the identification of *Fusarium* species to be *Fusarium temperatum* within the *Fusarium fujikuroi* species complex. No data were available in the *Fusarium* MLST database (http://www.cbs.knaw.nl/fusarium) for this isolate.

**Figure 3:**
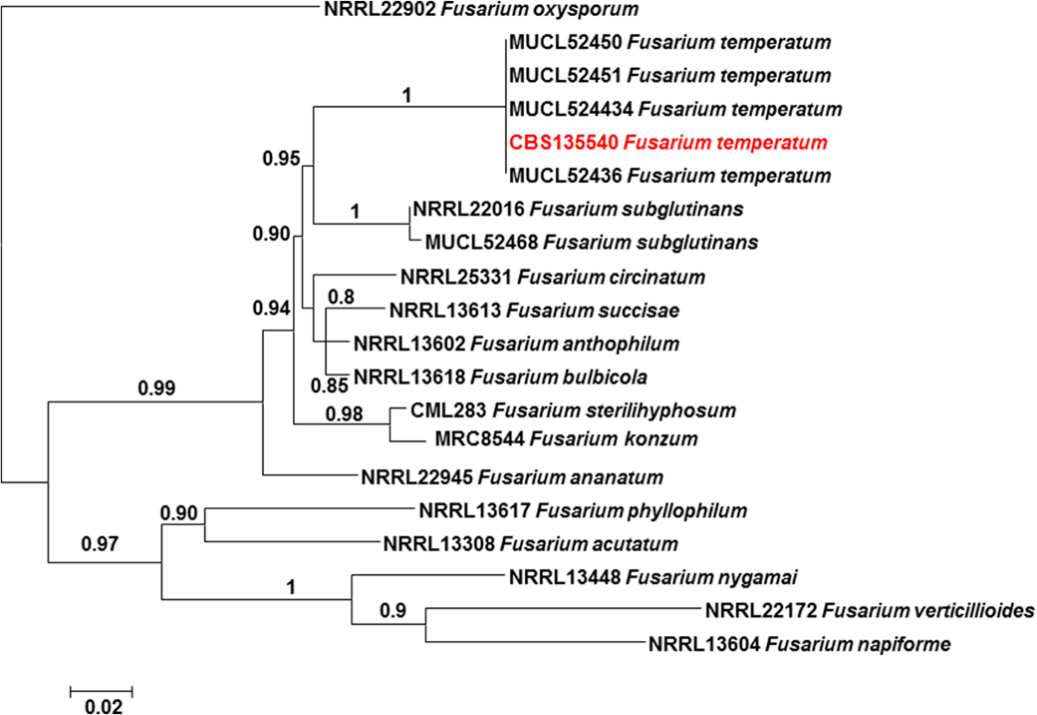
**Phylogenetic analysis of** ***Fusarium temperatum.*** Phylogenetic tree resulting from Bayesian analysis for the TEF-1α and β-tubulin genes (values of 0.8 for Bayesian probability are shown). *Fusarium oxysporum* (NRRL 22902) was used as the outgroup.

In order to establish the phylogenetic position of the *F. temperatum* clade, a general tree was made with MrBayes v. 3.1.2 on the Cipres Portal based on the BT2 (500 bp) and TEF-1α (600 bp) regions. Fifteen species within the *Fusarium fujikuroi* species complex clade were selected for phylogenetic analyses and sequences of the BT2 and TEF-1α genes were aligned among the sequences available from GenBank (Additional file 1: Table S1). Bayesian analysis was done by using Metropolis-coupled Markov chain Monte Carlo sampling approach to calculate posterior probabilities. Four simultaneous Markov chains, three heated and one cold, were run under a mixed model of sequence evolution and gamma approximation for rate variation among sites. Chains were analysed with random starting trees for 10^7^ generations, sampling from trees every 1000th generation. The burn-in period was set at 25%. Topologies of the trees generated with either gene (TEF-1α and BT2) were concordant. Partition Homogeneity Test (PHT = 0.97) did not detect conflict between loci, and therefore, these two genes were combined to investigate species delimitation using PSR (Figure 3). The BT2 and TEF-1α phylogenetic analyses showed that the reported clinical isolate was nested within the *Fusarium fujikuroi* species complex and was found to be identical to four environmental strains of *F. temperatum*.

### Antifungal susceptibility

Susceptibility testing was performed according to the guidelines of the Clinical and Laboratory Standards Institute document M38-A2. Our strain was highly susceptible to micafungin with an MIC of 0.031 μg/ml, followed by posaconazole with an MIC of 0.25 μg/ml and amphotericin B with an MIC of 0.5 μg/ml. Low MICs were also found for voriconazole (1 μg/ml) followed by isavuconazole, natamycin and anidulafungin, with MICs of 4 μg/ml. The azoles for which high MICs were found were fluconazole (>64 μg/ml) and itraconazole (>16 μg/ml) (Table 2).

**Table 2:** **MIC values of clinical isolate** ***Fusarium temperatum*** **, CBS135540**

Drug	AMB	FLC	ITC	VOR	POS	NATA	ISA	ANI	MICA
MIC values(μg/ml)	0.5	>64	>16	1	0.25	4	4	4	0.031

*AMB* Amphotericin B, *FLC* Fluconazole, *ITC* Itraconazole, *VOR* Voriconazole, *POS* Posaconazole, *ISA* Isavuconazole, *NATA* Natamycin, *ANI* Anidulafungin, *MICA* micafungin.

## Discussion

Mycotic keratitis is an important ophthalmologic problem with slow progression that must be distinguished from its bacterial counterpart [21] in view of appropriate treatment. Despite developments in diagnostics and therapy, the infection remains a significant public health problem, occasionally leading to significant visual disability [22]. *Fusarium* species are among the most common etiologic agents of the disorder [23]-[25] and are problematic because of their therapy-refractive nature. In direct microscopy *Fusarium* species are indistinguishable from *Aspergillus* because both produce hyaline, septate hyphae (Figure 1B), and therefore supplementary diagnostics are necessary.

For accurate identification of the causative agent, multi-locus analysis involving of parts of TEF-1α and BT2 genes, known to be informative at the species level in *Fusarium*[19],[26]-[28] was performed. Identification of the etiological agent as *F. temperatum* was unambiguous. The present case extends the significance of genus of *Fusarium* as agents of keratitis and underscores the utility of molecular methods in verification of these emerging fungi in the human host [29].

*Fusarium* species are distributed worldwide in a wide diversity of habitats such as soil, plant debris, and as pathogens on a wide diversity of plant hosts [30]. Some species synthesize mycotoxins, which may accumulate in infected plant tissue before harvest or in stored agricultural products where they can be harmful for humans [31]. *Fusarium* in agricultural products should not only be considered as a food spoilers, but also are a risk factor for farmers and harvesters dealing with infected farming material [32], causing traumatic infections.

A remarkable feature in *Fusarium* is the apparent combination of plant pathogenicity and the ability to cause infections in humans. A study of members of the *Fusarium solani* species complex demonstrated that strains from clinical specimens, sewage, and plants were all capable of infecting zucchini and growing at 37°C [33]. Zhang et al. [34] noted that a group of clinical isolates were identical to *F. solani f. sp. cucurbitae* race 2, which infects squashes [35]. Conversely, *F. oxysporum f. sp. lycopersici* race 2 killing tomato plants was also able to cause disseminated, fatal infection in a murine model [36]. Several studies reported *Fusarium subglutinans* as an important pathogen of maize, causing stalk and seeding malformation [36],[37], but the species has been reported as a human opportunist [38],[39].

Scauflaire et al. [40] examined the taxonomic status of 30 *Fusarium* strains isolated from maize fields in Belgium including three isolates named *F. subglutinans* and described them as a new species, *F. temperatum*[40]. Subsequently the authors [41] used different pathogenicity tests such as toothpick inoculation to obtain better understanding of the infection process in maize plants and concluded that *F. temperatum* is a host-specific plant pathogen. The species causes seedling blight chlorosis and forms necrotic lesions in maize stalks; in addition, the species produces mycotoxins. Pintos et al. [42] reported *F. temperatum* seedling malformations, chlorosis, shoot reduction and stalk rot in maize growing in inoculated soil.

The present case concerns a further example of a plant pathogen in addition to 70 other *Fusarium* species causing human infection. Such examples are expected to be rare, because in general degradation of plant versus animal components requires entirely different enzymatic machinery. Van Baarlen et al. [43] noted molecular similarity between hypothetical virulence factors in plant and human pathogens, but in practice such species are extremely uncommon [44].

Our patient developed a mycotic keratitis after traumatic introduction with maize leaves during harvest. Its clinical diagnosis was made by the ophthalmologist. At the clinical laboratory, it was first misdiagnosed as *F. oxysporum* using morphology. These results indicate that morphologic identification has some limitations.

Cardinal growth temperatures showed optimal development at 27°C (Figure 2), with a maximum growth temperature at 36°C. No growth was observed at 37°C and 40°C; these temperatures proved to be fungistatic as regrowth was observed after incubation at 25°C. The relatively low maximum growth temperature allows superficial infections such as keratitis. Our patient’s infection was first clinically diagnosed as a reactional or bacterial ophthalmitis, leading to the use of a preparation that contained two antibiotics, a decongestant (vasopressor), and a synthetic glucocorticoid. It is noteworthy to record that most cases of keratitis are treated on the basis of clinical features with antibiotics and steroids, which are compounds that stimulate fungal growth [45], while co-inoculation of a fungal agent is common. At observation of fungal hyphae in tissue, therapy was changed to natamycin and itraconazole.

The high susceptibility of CBS 135540 was unexpected because of the intrinsic resistance against antifungal drugs in *Fusarium* in general [46]-[48]. The *F. temperatum* isolates had low MICs for micafungin (0.031 μg/ml), posaconazole (0.25 μg/ml) and amphotericin B (0.5 μg/ml). Antifungal susceptibility testing proved that itraconazole and fluconazole were ineffective, and therefore clinical improvement of our patient was probably entirely due to natamycin because the present study shows that the natamycin had good activity against *F. temperatum* (4 μg/ml). Patient was not available for follow-up. In similar cases of keratitis, natamycin, anidulafungin, micafungin, voriconazole and amphotericin B were found to be effective [49].

## Conclusion

*Fusarium* species in general show high degrees of resistance to most antifungals. Using molecular identification to identify a number of rare medically important fungal genera and species, including *Fusarium*, is very important to predict therapeutic outcome and the emergence of these Fungi. Moreover, this paper provides evidence indicating *Fusarium temperatum* as a potential human opportunist that may have decreased susceptibility to azoles and other antifungal agents.

### Availability of supporting data

The data sets supporting the results of this article are available in the [LabArchives, LLC] repository, [http://dx.doi.org/10.6070/H46D5QZK].

The phylogenetic tree supporting the results of this article is available in the [TreeBASE] repository, [http://purl.org/phylo/treebase/phylows/study/TB2:S16415?x-access-code=ac517f778e8736faf6a6a3e9e4f1b263&format=html].

## Authors’ contributions

LVM collected the clinical data and patient’s history. KGC collected the clinical specimen and the clinical data. AB performed the phenotypic identification and helped with the draft of the manuscript. JFM performed the susceptibility test and helped with the draft of the manuscript. AMS performed molecular identification, phenotypic description and wrote the manuscript. ADvD helped with the draft of the manuscript. SdH supervised the coordination and concept of the manuscript. All authors have read and approved the manuscript.

## Additional file

## Electronic supplementary material

Additional file 1: Table S1.: Strains features used for phylogeny. Collection, origin, and GenBank accession numbers used in the present study for phylogenetic analysis of *Fusarium temperatum*, CBS 135540. (DOCX 15 KB)

Below are the links to the authors’ original submitted files for images.

Authors’ original file for figure 1

Authors’ original file for figure 2

Authors’ original file for figure 3

## Abbreviations

TEF1 α: Translation elongation factor 1 alpha
BT2: Beta-tubulin
MLST: Multilocus sequence typing
MEA: Malt extract agar
OA: Oatmeal agar
PDA: Potato dextrose agar
SNA: Synthetic nutrient agar
CLA: Carnation leaf agar
PCR: Polymerase chain reaction
MIC: Minimum inhibitory concentration
AMB: Amphotericin B
FLC: Fluconazole
ITC: itraconazole
VOR: Voriconazole
POS: Posaconazole
NATA: Natamycin
ISA: Isavuconazole
ANI: Anidulafungin
MICA: Micafungin
MEC: Minimal effective concentrations
