# Supplementary material for: Keratitis by Fusarium temperatum, a novel opportunist
Source: BMC Infect Dis. 2014 Nov 12;14:588. doi: 10.1186/s12879-014-0588-y (PMC4234859; doi:10.1186/s12879-014-0588-y)
Supplement: Supplementary file 1 — Additional file 1: Table S1.: Strains features used for phylogeny. Collection, origin, and GenBank accession numbers used in the present study for phylogenetic analysis of Fusarium temperatum, CBS 135540. (DOCX 15 KB) [file 12879_2014_588_MOESM1_ESM.docx]

**Additional file 1: Table S1 - Strain features and GenBank accession numbers used in the present study for phylogenetic analysis of *Fusarium temperatum*, CBS 135540**

| **Species** | **collection** | **Origin** | **β -tubulin** | **TEF 1α** | **Refe** |
| --- | --- | --- | --- | --- | --- |
| *F. verticillioides* | NRRL 22172 | Germany | U34413 | AF160262 | [40] |
| *F. napiforme* | NRRL 13604 | South Africa | U34428 | AF160266 | [40] |
| *F. nygamai* | NRRL 13448 | Australia | U34426 | AF160273 | [40] |
| *F. acutatum* | NRRL 13308 | India | U34431 | AF160276 | [40] |
| *F. phyllophilum* | NRRL 13617 | Italy | U34432 | AF160274 | [40] |
| *F. subglutinans* | NRRL 22016 | USA | U34417 | AF160289 | [40] |
| *F. subglutinans* | MUCL 52468 | Belgium | HM067699 | HM067691 | [40] |
| *F. circinatum* | NRRL 25331 | USA | U61547 | AF160295 | [40] |
| *F. bulbicola* | NRRL 13618 | Netherlands | U61546 | AF160294 | [40] |
| *F. anthophilum* | NRRL 13602 | Germany | U61541 | AF160292 | [40] |
| *F. succisae* | NRRL 13613 | Germany | U34419 | AF160291 | [40] |
| *F. temperatum* | MUCL 52450 | Belgium | HM067695 | HM067687 | [40] |
| *F. temperatum* | MUCL 52436 | Belgium | HM067692 | HM067684 | [40] |
| *F. temperatum* | MUCL 52443 | Belgium | HM067693 | HM067685 | [40] |
| *F. sterilihyphosum* | CML 283 | Brazil | DQ445780 | DQ452858 | [40] |
| *F. konzum* | MRC 8544 | USA | EU220234 | EU220235 | [40] |
| *F. ananatum* | NRRL 22945 | England | U34420 | AF160297 | [40] |
| *F. oxysporum* | NRRL 22902 | USA | U34424 | AF160312 | [40] |
| *F. temperatum* | CBS135540 | Mexico | KF956084 | KF956080 | This study |
