# Supplementary figures and images for: Keratitis by Fusarium temperatum, a novel opportunist
Source: BMC Infect Dis. 2014 Nov 12;14:588. doi: 10.1186/s12879-014-0588-y (PMC4234859; doi:10.1186/s12879-014-0588-y)

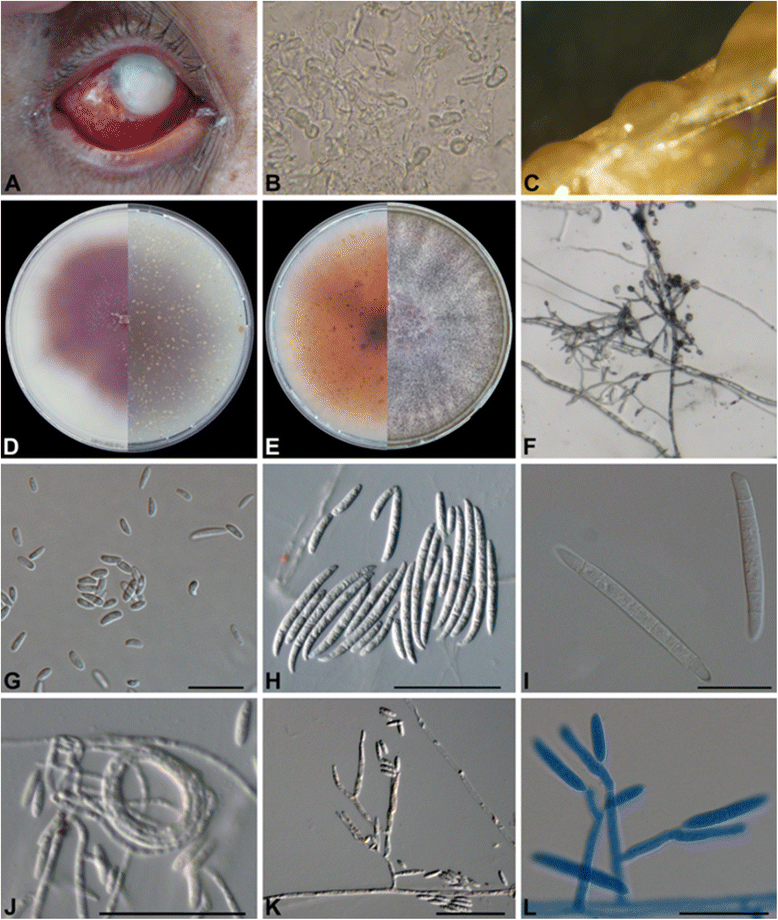

Supplement: Supplementary file 2 — Authors’ original file for figure 1 [file 12879_2014_588_MOESM2_ESM.gif]

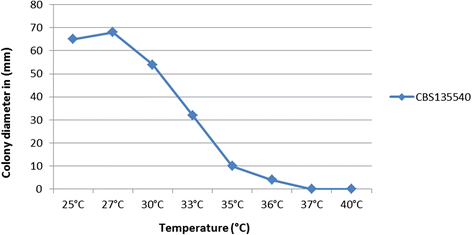

Supplement: Supplementary file 3 — Authors’ original file for figure 2 [file 12879_2014_588_MOESM3_ESM.gif]

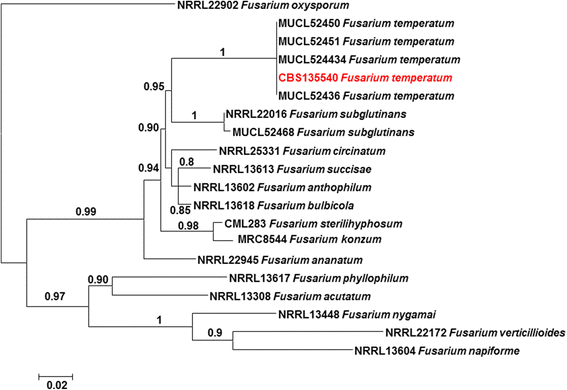

Supplement: Supplementary file 4 — Authors’ original file for figure 3 [file 12879_2014_588_MOESM4_ESM.gif]
